# Advances and Applications of Hollow Fiber Nanofiltration Membranes: A Review

**DOI:** 10.3390/membranes11110890

**Published:** 2021-11-19

**Authors:** Tim Sewerin, Maria G. Elshof, Sonia Matencio, Marcel Boerrigter, Jimmy Yu, Joris de Grooth

**Affiliations:** 1 NX Filtration, Josink Esweg 44, 7545 PN Enschede, The Netherlands; t.sewerin@nxfiltration.com (T.S.); m.elshof@nxfiltration.com (M.G.E.); 2 LEITAT Technological Center, C/Pallars, 179-185, 08005 Barcelona, Spain; smatencio@leitat.org (S.M.); mboerrigter@leitat.org (M.B.); 3 Pepsi Co., Inc., Global R & D, 350 Columbus Ave, Valhalla, NY 10595, USA; jimmy.yu@pepsico.com; 4 Membrane Science & Technology, MESA+ Institute for Nanotechnology, University of Twente, P.O. Box 217, 7500 AE Enschede, The Netherlands

**Keywords:** hollow fiber, nanofiltration membrane, industrial applications

## Abstract

Hollow fiber nanofiltration (NF) membranes have gained increased attention in recent years, partly driven by the availability of alternatives to polyamide-based dense separation layers. Moreover, the global market for NF has been growing steadily in recent years and is expected to grow even faster. Compared to the traditional spiral-wound configuration, the hollow fiber geometry provides advantages such as low fouling tendencies and effective hydraulic cleaning possibilities. The alternatives to polyamide layers are typically chemically more stable and thus allow operation and cleaning at more extreme conditions. Therefore, these new NF membranes are of interest for use in a variety of applications. In this review, we provide an overview of the applications and emerging opportunities for these membranes. Next to municipal wastewater and drinking water processes, we have put special focus on industrial applications where hollow fiber NF membranes are employed under more strenuous conditions or used to recover specific resources or solutes.

## 1. Introduction

While nanofiltration (NF) membranes have been available since early 1980 [1] only more recently has the attention to these membranes for water treatment processes started growing increasingly, both in academia and in industry. The quality and availability of freshwater sources is becoming a major concern, and this has been included within sustainable development goals (SDGs) by the United Nations (UN), namely SDG 6: Ensure availability and sustainable management of water and sanitation for all. NF membranes offer a semi-selective barrier with selectivities that lie between the dense reverse osmosis (RO) membranes and porous ultrafiltration (UF) membranes. Where RO membranes can retain even the smallest solute to desalinate seawater, UF membranes allow for efficient removal of bacteria, viruses, and total suspended solids (TSS) and NF membranes enable the selective removal of specific solutes or the purification of water at lower energy consumption [1,2]. This semi-permeability of NF also hits the sweet spot within the filtration spectrum for many emerging processes.

NF membranes have been categorized as membranes with effective pore diameters between 1 and 5 nm [3]. This means that these membranes cover the transition from convective flow through distinct physical pores to solution and diffusion in a dense polymer network. Different models have been developed to describe the transport through these membranes [4,5,6,7], but their applicability will depend on both the membrane and the solute. The versatility that NF offers also means that there is a wide variation in the possible applications and processes. Several more obvious applications, such as municipal wastewater filtration, have received great notice lately [8], but attention on other specific industrial applications is still much more scattered. However, these industrial applications do have the potential of being a main driving force for the growth and acceptance of novel NF membranes.

**Figure 1:**
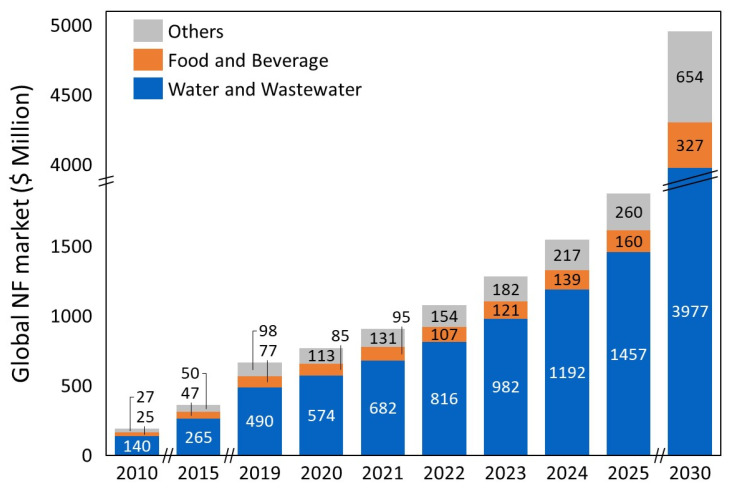
Global annual NF market per application in $ million, including the projected growth for 2020–2030 [9].

Currently, with an estimated share of 7%, NF membranes play only a minor part in the total membrane market [9], but this NF market is growing faster compared to conventional membrane technologies (e.g., UF and RO). This is partially attributed to the global increase in demand for potable water, where NF membranes can provide an environmentally friendly and cheaper alternative. Moreover, a substantial part of the projected growth lies in the aforementioned new industrial applications (see Figure 1). 

Several different membrane geometries are available for NF membranes. Until recently, predominantly spiral-wound (SW) configurations were commercially available. These types of membranes are comparable to the denser RO modules used for desalination. Typically, the membranes consist of a porous support on which a thin polyamide layer is applied via interfacial polymerization (IP). By using monomers such as piperazine (PIP) [10] a relatively more hydrophilic and open NF membrane is obtained as compared to RO membranes. An advantage of the SW membranes is that the configuration uses the same standard housings, connections, and pressure vessels as RO systems, potentially reducing the total capital expenditures of the total system for the end-user. In addition, due to similarities to the high-pressure RO configuration, these SW NF membranes can also be operated at relatively high pressures [1]. However, the SW configuration necessitates an extensive pre-treatment of the feed, such as UF or sand filtration (SF). The SW configuration cannot handle high levels of suspended solids, as the membranes are prone to clogging and susceptible to (bio)fouling. The latter is especially of concern as the polyamide chemistry of the selective NF layer is not stable in standard oxidizing chemical solutions [11] (preferably based on NaOCl or H_2_O_2_), and thus special biocides need to be applied to remove the biofouling [12].

More recently, hollow fiber (HF) NF membranes have become commercially available. An overview of current manufacturers of HF NF membranes and their products is shown in Table 1. The HF configuration can have a large surface area to volume ratio [13,14,15], albeit this is dependent on the fiber diameter. HF NF membranes can handle feed streams with much higher levels of suspended solids, matching or even surpassing UF membranes. In addition, the possibility of hydraulically cleaning the membranes, such as via backwashing [16] and air sparging [17], further enables the filtration of fouling prone feed waters, reducing the necessity of an extensive pre-treatment [18,19]. Nevertheless, biofouling is a potential threat for these membranes and therefore several of the commercially available HF NF membranes have been developed based on selective layers that are chemically stable. This, for instance, allows the use of oxidizing chemical solutions [18,20]. This combination of chemical stability and HF geometry has been a big accelerator for the development of these new membranes, as they enable simple and direct water treatment processes based on NF-like selectivities. In comparison to SW NF membranes, the operating pressures of the commercial HF NF membranes are substantially lower. This stems typically from a specific operational expenditure perspective [2,21], but also, at high operating pressures the fiber integrity is at risk, which can be detrimental to the filtration efficacy [22]. The lower operating pressures can result in a reduced specific energy consumption during operation as compared to SW NF and RO membranes.

This review paper contains a brief summary of the HF NF membrane preparation methods and then focuses on covering a non-exhaustive overview of different (emerging) applications of HF NF membranes. Special attention is given to the applicability of HF NF membranes in industrial applications, where both existing applications (published in the last 10 years) and emerging processes will be discussed.

## 2. Preparation Methods

**Figure 2:**
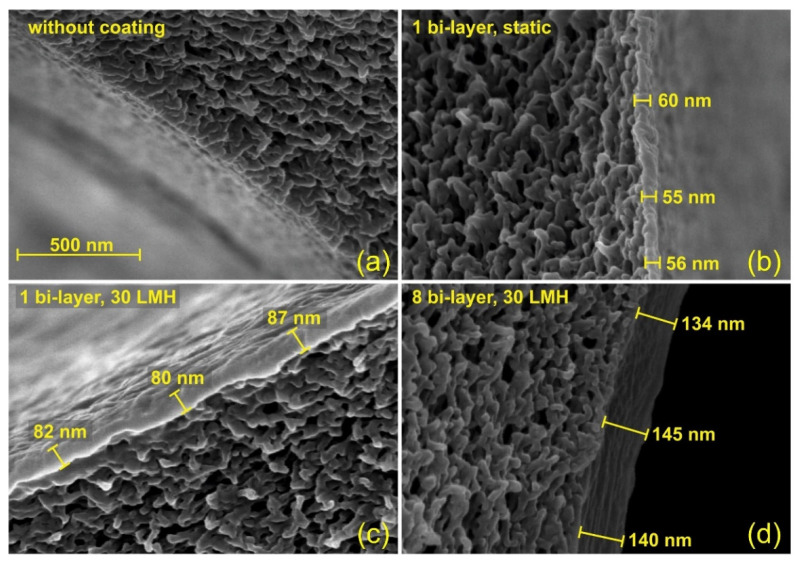
Scanning electron microscopy images of 4 different membranes: (**a**) Without coating, (**b**) 1 bilayer coated in static mode with an adsorption time of 10 min, (**c**) 1 bilayer coated in dynamic mode at 30 LMH for 10 min, (**d**) 8 bilayers coated in dynamic mode at 30 LMH for 10 min. Reprinted from [38], with permission from Elsevier.

The selective layer of HF NF membranes can be prepared via a variety of methods, that can roughly be divided into five different categories: Directly during phase inversion, polymerization, coating, grafting and self-assembly [28]. Phase inversion is one of the key methods to prepare the HF support, but can also be used to create the selective NF layer during fiber spinning [29]. Phase inversion involves the separation of a polymer solution into a polymer-rich and a polymer-lean phase, leading to a porous solid. For HF membranes, the polymer solution is extruded through a double or triple orifice spinneret and phase separation is usually non-solvent induced, where the polymer solution is extruded in a coagulation bath. Co-extrusion via a triple orifice, solvent evaporation or chemical reactions and complexations during phase separation can be employed to yield dense layers directly [30,31,32,33]. Alternatively, a post-treatment of the porous fiber is needed to form the selective layer. Interfacial polymerization (IP) is a polymerization method where two reactive monomers react at the interface of two immiscible solvents and form a thin polymeric film [28]. The IP is performed on top of a porous support, which results in a thin-film composite (TFC) NF membrane. Dip-coating is a very simple method to apply a thin film, of which the final properties can be tailored [34]. Grafting can be used to prepare HF NF membranes: UF membranes can be chemically modified via graft polymerization by different methods, such as plasma, E-beam, UV/photo and γ-ray irradiation to result in charged HF NF membranes [35,36]. Finally, a successful method is the preparation of an NF layer via the self-assembly of polycations and polyanions to form polyelectrolyte multilayers (PEM) on a porous support [37], often via a Layer-by-Layer (LbL) type of approach. This method has gained attention recently because of its ease of fabrication, tunability and relatively sustainable preparation method and different methods of applying the PEM have even been developed to obtain controllable thin layers, see Figure 2 [38]. These advantages of the PEM have resulted in the commercialization of these types of membranes.

## 3. Applications

In the following subsections an overview is provided of both existing and emerging applications for HF NF membranes. For this, a distinction has been made between applications where the aim is to recover or purify freshwater, i.e., where water is the valuable product, and applications where the goal is to recover valuable solutes present in (aqueous) streams. The examples mentioned are meant to show the potential of HF NF membranes; however, the list is not meant to be exhaustive.

### 3.1. Freshwater Treatment

#### 3.1.1. Natural Organic Matter Removal

Natural organic matter (NOM) is ubiquitous in natural aquatic environments like rivers, lakes and marine systems. However, NOM has a significant impact on drinking water production as it leads to poor water aesthetics (color, taste and odor), forms hazardous disinfection by-products like trihalomethanes by reacting with chlorine, and serves as a carbon and energy source for microbial fouling and regrowth in water distribution systems [39]. In recent decades, increased NOM concentrations have been reported in America, Europe and Asia [40]. Hence, dealing with elevated NOM levels will be a future challenge for drinking water systems. NFs can be an effective barrier for NOM due to their typical molecular weight cut-off (MWCO) of 200–1000 Da [41]. Applications for SW membranes have been reviewed extensively elsewhere [42]; however, the implementation of NF is still limited. A main reason for this is that NOM contributes significantly to membrane fouling [39]. This is especially an issue for spiral-wound modules, which are prone to (bio)fouling and have limited cleaning possibilities [12]. HF NF membranes have been named as a promising alternative [20,43], since they offer the advantage to combine the beneficial cleaning conditions of HF UF membranes with the separation efficiency of spiral-wound modules.

Köhler et al. investigated the performance of commercially available HF NF membranes (HFW 1000, Pentair X-Flow) on NOM removal by running a pilot installation on lake water in Sweden [44]. It was found that a combined process of coagulation and HF NF effectively removed 90% of the dissolved organic carbon (DOC) and 96% of the UV absorbance, while less than 20% of the hardness was retained. Biopolymers and humic substances were almost completely removed. An autopsy of the membrane was performed after 12 months of operation, which demonstrated no significant changes compared to the virgin membrane [20]. In a follow-up pilot study, the same membrane type was evaluated for drinking water production on the same lake water without the prior coagulation [45]. At the optimal process conditions (crossflow velocity of 0.75 m·s^−1^, 80% recovery and flux between 12 and 18 L·m^−2^h^−1^), the UV absorbance was reduced by 80%; 75% of the humic substances (measured molecular weight (MW): 530–660 Da) were removed as well as 70% of total organic carbon (TOC) in only one step. However, only 40% of the low molecular weight acids (MW = 300–400 Da) were removed from the water. Lidén et al. tested the commercial HF NF (HFW 1000, Pentair X-Flow) in a pilot installation on NOM removal for three different surface waters from boreal lakes in Sweden. A TOC retention of 88% and a UV absorbance retention of 93% were reported [46]. All these results show the potential of HF NF membranes for the treatment of (surface) water where mainly NOM needs to be removed. High removal rates are achieved, which can even be obtained without the addition of coagulants or other pre-treatment steps. 

Indeed, next to these pilot studies, commercial installations using HF NF for NOM removal are already in operation or under construction. The HFW 1000 membranes from Pentair X-Flow are applied in a full-scale membrane system on surface water in Tasmania, Australia. The water treatment plant provides 60 m^3^·h^−1^ of potable water in a chemical-free one-step process [47]. Currently, a water treatment plant in Indonesia is being upgraded with HF NF dNF80 membranes from NX Filtration to remove NOM from peat water for drinking water production [48]. During pilot study tests, HF NF proved their suitability to achieve drinking water quality in one step, while enabling a chemical-free operation. Initially, a first stage providing a capacity of 180 m^3^·h^−1^ is built, which will be expanded to 1620 m^3^·h^−1^ in three stages. In an extensive study by Aggarwal, the environmental impact of the HF NF process was compared to more conventional water treatment schemes (based on combinations of, e.g., precipitation, sedimentation processes, rapid sand filtration and UF) [49]. The analysis shows that the HF NF process was the most sustainable alternative, mainly resulting from the low chemical use during operation.

#### 3.1.2. Water Softening

Hardness removal, which is often referred to as water softening, is an important step in water treatment. A high level of hardness, which is mainly determined by the presence of Ca^2+^ and Mg^2+^, can cause scaling problems in plumbing, heat exchangers or boilers. This will decrease the efficiency of heat exchangers leading to increased energy cost and cleaning effort [50]. Therefore, it is beneficial to reduce high hardness levels. Conventional NF membranes are well established for this due to their efficient removal rates for multivalent ions [51]. However, SW NF modules are suspectable to scaling due to the spacer configuration [52]. The HF NF geometry with a smooth inner surface can offer a significant improvement for the scaling issue. It enables the application of diverse cleaning methods as well as mitigating the scaling build-up due to its smooth surface. 

Fang et al. developed interfacially polymerized composite HF NF membranes using branched polyethyleneimine (PEI) and trimesoylchloride (TMC) as the monomers on a polyethersulfone (PES) UF membrane support and investigated the performance for water softening [53]. Artificial hard water was created with an overall total dissolved solids (TDS) load of 3000 ppm, mimicking a brackish water source in Florida. At a water flux of 20 L·m^−2^h^−1^ at 2 bars of feed pressure, the membrane successfully rejected 84.8% and 81.0% of Mg^2+^ and Ca^2+^, respectively, while Na^+^ retention was as low as 14.3%. Fang et al. then investigated the performance of a PEI/PIP mixed composite HF NF membrane for hardness removal [54]. The membrane was tested on artificial water with a TDS load of 3000 ppm and an overall hardness of 691 mg·L^−1^ as CaCO_3_. Hardness removal of over 90% was reported, whereas Na^+^ rejection was as low as 18%. The tests were conducted at 2 bars pressure and a flux of 12.8 L·m^−2^h^−1^ was achieved. Effective hardness removal from artificial waters on a lab-scale has been reported for other HF NF membranes as well [21,55]. 

Recently, Sengur-Tasdemir et al. investigated various HF NF membranes’ performances during pilot-scale filtration tests of surface water from a lake reservoir in Turkey. The reinforced thin-film composite membrane showed the best results regarding water softening as a hardness removal of 57% was achieved [56]. A constant flux of 4.76 L·m^−2^h^−1^ was reported over the testing duration of 150 h.

### 3.2. Municipal Wastewater Treatment

Municipal wastewater treatment focusses on the removal of nutrients and contaminants from wastewater originating from households and businesses. The water streams are traditionally treated by a wastewater treatment plant (WWTP), that consists of several different steps, to produce an effluent that is safe to discard. However, regulations for discharge are becoming stricter for a variety of components and not everything can be sufficiently removed by traditional WWTPs. Membrane filtration offers a possible solution to produce effluent streams that will meet the more stringent disposal limits [57].

#### 3.2.1. Organic Micropollutant Removal

The worldwide occurrence of organic micropollutants (OMPs), such as pharmaceuticals, personal care products, hormones, surfactants, industrial chemicals and pesticides, in water bodies and municipal and industrial wastewater streams is creating more and more concern [58]. Due to their ecotoxicological impact and potential risk for human health, further treatment steps are necessary to upgrade WWTPs, which are a main entry source for OMPs in the environment [59,60].

Some of the first results on OMP removal with LbL-based HF NF membranes were published by de Grooth et al. in 2014 [61]. A HF UF support was coated with polyelectrolyte trilayers, consisting of poly(diallyldimethylammonium chloride) (PDADMAC) as polycation, poly N-(3-sulfopropyl)-N-(methacryloxyethyl)- N,N-dimethylammonium betaine (PSBMA) as polyzwitterion and poly(styrenesulfonate) (PSS) as polyanion. Filtration tests on six different OMPs with molecular weights of 215 to 360 g·mol^−1^ resulted in high retention for positively and negatively charged OMPs, while relatively low retentions for uncharged OMPs were reported. 

Using the same HF UF support, Ilyas et al. coated weak polyelectrolyte multilayers (PEMs) using poly(allylamine hydrochloride) (PAH) and poly(acrylic acid) (PAA) while varying the pH conditions of the coating steps [62]. Coating at a pH of 6.0 resulted in retentions of 60–80% for charged (positively and negatively) as well as neutral OMPs and at the same time allowing a high salt passage. However, the obtained permeability was rather low at 1.8 L·m^−2^h^−1^bar^−1^. To further evaluate the membrane’s performance in realistic wastewater conditions, Abtahi et al. tested the same membrane on synthetic wastewater containing four OMPs (diclofenac, naproxen, ibuprofen and 4n-nonylphenol) in representative concentrations [63]. Measured OMP retentions ranged from 44% (ibuprofen) to 77% (diclofenac) and therewith corresponding with previous data, while the reported NaCl retention was as low as 17%, confirming a potential for OMP removal without producing a saline concentrate.

In 2019 Abtahi et al. investigated the impact of salt annealing post-treatment of PAH/PAA PEM flat-sheet membranes on OMPs retention [64]. The salt-annealed PEM membranes showed superior rejection rates for the tested compounds diclofenac, naproxen, ibuprofen and 4n-nonylphenol (52–82% against 43–69%) in comparison to non-annealed PAH/PAA membranes. At the same time, the ion rejections were moderately low with a NaCl retention of 25%. Furthermore, the pure water permeability was measured as 11.8 L·m^−2^h^−1^bar^−1^, demonstrating no decrease due to salt annealing. Next to that, the applicability of the so-called sacrificial layer approach for fouling removal was investigated by removing and subsequent recoating of the PEMs. The coated multilayers could be entirely removed, or sacrificed, and after recoating a permeability identical to the pristine coated membrane was achieved. In contrast to other studies on sacrificial layers [65], a complete removal of foulants without the necessity to employ physical force was obtained, demonstrating the beneficial effect of the sacrificial layer approach. As municipal wastewater contains a relatively high load of organics, fouling control is an important parameter for stable operation, which makes this approach interesting for applications in wastewater treatment.

**Figure 3:**
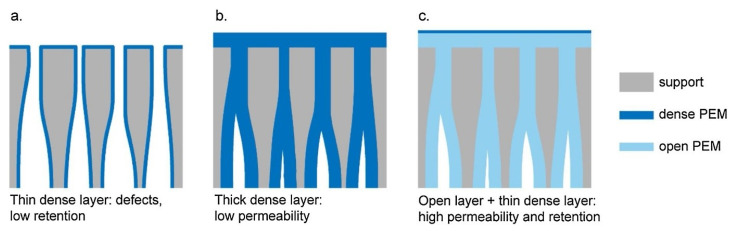
Concept of asymmetric PEM membranes. (**a**) Coating a thin dense layer on a porous support will lead to defects; (**b**) if the support pores are filled with a dense PEM with good separation properties, water needs to travel a long pathway through the PEM and the water permeability will be low; (**c**) if the support pores are filled with an open PEM, and a thin layer of dense PEM with the desired separation properties is coated on top, a combination between a high selectivity and permeability can be achieved. Reprinted from [66], with permission from Elsevier.

More recent research on LbL membranes for OMP removal focused on asymmetrical PE layers by combining open and dense PEMs. With this approach, pores are closed, and defects are eliminated by the relatively thick but highly permeable PEM, while high retention is ensured by the thin but dense PEM top layer (Figure 3). Te Brinke et al. achieved a novel type of membrane by coating a porous support with an open PSS/PAH multilayer followed by an ultra-thin top layer of PAA/PAH bilayers with a resulting thickness of 4 nm [66]. Investigation of the salt and OMP removal of this asymmetrical membrane demonstrated an average retention of 98% for in total nine charged and uncharged OMPs, with MWs ranging from 216 to 624 g·mol^−1^, while the NaCl removal was around 10–15%. These results confirm an outstanding permselectivity regarding salt permeation over OMP permeation combined with a permeability as high as 12.8 L·m^−2^h^−1^bar^−1^.

As follow-up research, the same research group tested different production techniques and alternative polyelectrolytes to assemble asymmetrical PEMs [67]. A porous HF support was coated with an open PSS/PDADMAC PEM and different processes for creating a dense top layer were investigated. While variation of the ionic strength in the top layer coating did not have significant improvements in comparison to a purely PSS/PDADMAC coated membrane (52% average OMP retention), adding a PSS/PAH PEM top layer at high ionic strength instead led to an average OMP removal as high as 93% and a pure water permeability of 5.9 L·m^−2^h^−1^bar^−1^. The NaCl retention was around 50%. Moreover, additionally cross-linking the top layer with glutaraldehyde further increased the OMP retention to 98% on average along with a slight decrease of permeability by 20%. While the achieved separation is equal to the previously published data at lower permeability [66], the cross-linking is predicted to be more chemical resistant, allowing usage for harsher conditions and cleaning procedures and therewith showing the high potential for OMP removal. 

Lately, a new type of PEM membrane for OMP removal was produced. In 2020, Li et al. immobilized laccase in between PSS/PAH multilayers and simultaneously on the dense separation layer achieving LbL-based biocatalytic NF membranes [68]. Post immobilization appeared to be the favorable production strategy, resulting in a competitive pure water permeability of 10.9 L·m^−2^h^−1^bar^−1^ and laccase loading and activity. A bisphenol (BPA) removal of 92.5% was achieved and found to be robust, maintaining the high retention after six cycles within 14 days.

The high retentions of micropollutants by HF NF membranes are not only limited to LbL PEM membranes. Bolong et al. fabricated PES HF NF membranes blended with charged surface modifying macromolecules [69]. In 2015 Liu et al. showed that thin-film nanocomposite HF membranes could be prepared via dioxane assisted interfacial polymerization [70]. The SAPO-34 nanoparticle incorporated membranes showed pure water permeability of 20.1 L·m^−2^h^−1^bar^−1^ and high rejection (>96%) for the micropollutants tris(2-chloroethyl) phosphate, tris(1-chloro-2-propyl) phosphate and tris(1,3-dichloro-2-propyl) phosphate.

A special type of micropollutant are the so-called “forever chemicals”, compounds that do not naturally break down. Per- and polyfluoroalkyl substances (PFAS) are hydrocarbons in which either all or most hydrogen atoms on the alkyl chain are replaced by fluorine atoms. The most well known examples are perfluorooctane sulfonate (PFOS) and perfluorooctanoic acid (PFOA). They are used in a wide variety of applications because of their unique physico-chemical properties such as durability and ability to repel both water and oil [71]. Over the last years, concerns have been raised about PFAS, because they were shown to be both toxic and bioaccumulative. Because of this, PFOS and PFOA, among others, have been categorized as persistent organic pollutants (POP) [72]. PFAS are detected in surface, ground and tap water and cause a threat to the secure water. While there are countries that set regulations to limit the use of PFAS, it is still widely used because of a lack of alternatives. Therefore, research focuses on alternative water treatment methods to remove PFAS from water, as the conventional biological water treatment plants do not suffice.

The efficacy of the traditional SW NF membranes for the removal of PFAS was shown on several occasions. Soriano et al. showed the successful use of NF270 membranes for the removal of perfluorohexanoic acid from industrial process waters up to 99.4% [73]. The concentrated retentate stream could be treated separately with an electrochemical cell, leading to a TOC reduction of >95%. In a recent study, Zhao et al. investigated PFOS removal in the presence of humic acids and inorganic ions during NF with a commercial ESNA1-K1 membrane (Hydranautics) [74]. The presence of humic acids, cations and anions improved the PFOS removal up to 99% retention. 

Wang et al. showed that by using a poly(m-phenylene isophtalamide) HF NF membrane, trace amounts of PFOS could be rejected from an aqueous solution [75]. PFOS rejections of 91.17 to 97.49% were observed, with pH increasing from 3.2 to 9.5. With an increased concentration of Ca^2+^ in the feed solution, the rejection could be even further enhanced. It is expected that in the removal of PFAS from water sources, NF membranes can play a big role in securing the quality of water and both aquatic and human health.

#### 3.2.2. Antimicrobial Resistance

Antimicrobial resistance (AMR) is an emerging phenomenon as a result of the widespread use of antimicrobial agents. The occurrence can be enhanced via different pathways such as via WWTPs, overuse of antibiotics in aquaculture or animal manure in agriculture. The increased resistance against antibiotics can have a negative effect on both human and animal health. Therefore, it is considered as a global health threat according to the world health organization (WHO), UN and European Union (EU), which needs to be addressed [76]. 

AMR is transported by antibiotic resistant bacteria (ARB) and expressed by antibiotic resistant genes (ARGs) [77]. It is important to tackle the AMR issue at the source and ensure that antibiotics are not disposed into wastewater from where they can be discharged into the environment. NF can play a significant role in this by retention of antibiotics from hospital and industrial wastewater streams. The applicability of NF for this is already covered in the previous paragraph on micropollutants. However, AMR intervention consists of more than just micropollutant removal. While the bacterias might be handled with microfiltration (MF)/UF, resistant genes require a much lower cut-off, which open NF can provide [78]. Lan et al. investigated the use of NF for the removal of ARGs in swine wastewater. Removal rates of 5–8 log could be obtained for various kinds of ARGs with a polyamide NF membrane. However, these reduction values were calculated for the whole train of the WWTP. A range of membranes, ranging from MF to RO, were studied by Slipko et al. for the purpose of free DNA containing ARG removal [79]. Up to 99.0% of free DNA could be removed by NF membranes. Furthermore, Krzeminski et al. showed that open NF membranes with MWCO < 1 kDa (polyamide TFC) were effective for the complete removal of cell free DNA [76]. The obtained concentrate stream could in turn be treated by UV. Recently, Cristóvão et al. tested the use of Desal 5DK membranes at a domestic wastewater treatment plant [80]. They observed stable permeance, with high rejections for target antibiotics, ARGs and viral genomes. These examples provide promising results for potential application of NF in antibiotic removal. However, the number of publications on the removal of ARGs by NF is still limited and should gain more attention in order to solve the AMR issue. Results show that several NF membranes, with a wide range of cut-off and different geometries, can be employed and the best choice will rely on both the target compounds that need to be removed and the complete water matrix that needs to be treated.

#### 3.2.3. Micro- and Nanoplastics Removal

Roughly ten percent of the total amount of plastic produced eventually ends up in the water environment [81]. Since most plastics are not biodegradable, they accumulate in the aquatic environment and contribute to plastic pollution. The plastic fragments differ in sizes; typically, microplastics are particles smaller than 5 mm and nanoplastics smaller than 1 µm. These plastics fragments can have a negative impact on the aquatic environment [81,82], e.g., on growth, reproduction and mortality of aquatic animals. Next, potential negative effects to human health are described, e.g., the microplastic polystyrene has shown oxidative stress to human cerebral and epithelial cells [83]. Nanoplastics are considered even more dangerous, because of their small size and large surface area and the fact that they are more difficult to remove in conventional water-treatment processes. In recent years more focus has been put on how to remove these plastics from wastewater. Wastewater treatment plants are able to remove 75% of microplastics in the pre-, primary and secondary treatment steps. By using a tertiary treatment, the removal can be increased to 98% [84]. However, plastics smaller than 20 µm and nanoplastics are not removed. Therefore, improvements are still needed to also remove these compounds from the water. Malankowska et al. suggested that NF might be an efficient method for this purpose [85]. To the best of our knowledge, no research studies are available yet regarding the effectiveness of NF for this purpose, but it is expected to be evaluated in due time. In research by Trzaskus et al. it was shown that already with MF nanoparticles much smaller than the membrane pore sizes could be retained [86], e.g., 95% of 92 nm nanoparticles could be separated. Therefore, NF membranes are expected to be useful for the retention of even smaller nanoplastics.

#### 3.2.4. Wastewater Reuse

**Figure 4:**
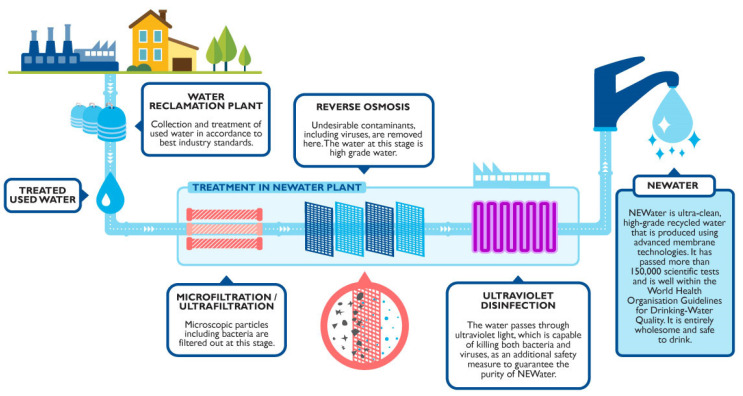
Schematic representation of the NEWater process to recycle treated used water into ultra-clean water [87]. Reprinted with permission from PUB, Singapore’s National Water Agency.

The aforementioned applications show the promising potential of HF NF membranes for municipal wastewater purification. As the water quality of the permeate will typically not only meet, but exceed the discharge limits, the high-quality treated water can potentially be reused in various applications, e.g., as process or boiler feed water in the industry, for agricultural irrigation, or even as potable water. In contrast to other water sources (e.g., surface water), municipal wastewater is mostly independent from seasonal weather variability and droughts, which enables a shift towards a more circular water economy. In Singapore, five NEWater plants are operated to reclaim pre-treated wastewater for reuse, supplying 40% of the country’s total water needs [87]. The three-staged NEWater process includes MF/UF, followed by an RO installation and a UV disinfection step (see Figure 4). The achieved water quality allows for use in wafer fabrication plants, where even stricter standards than for drinking water are required. During dry weather, this reclaimed water is also blended to raw water reservoirs.

**Figure 5:**
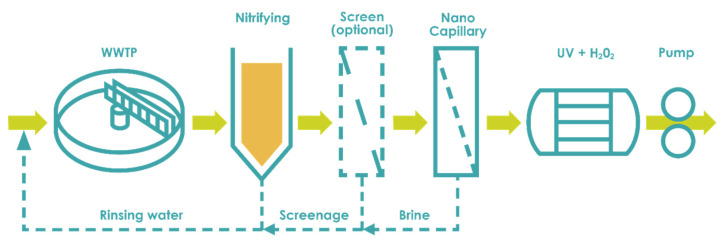
Schematic illustration of water factory water reuse case. Reprinted from [88] with permission from Energy and Resources Factory.

Similarly, HF NF could be applied to combine both membrane processes into one step. As has been shown before, HF NF membranes are able to deliver a water quality that allows for drinking water production or reuse in various industries. A wastewater reuse case using HF NF is proposed by the Dutch network organization Energy and Resources Factory. As shown in Figure 5, the WWTP’s effluent enters the HF NF directly after a denitrifying sand filter and provides a level of quality adhering to the Dutch Drinking Water Decree that is suitable for use as process water in most industries [88]. With an additional UV + H_2_O_2_ installation as a second disinfection step, total costs (capitalization and operational costs) of 0.50 €·m^−^^3^ were reported, which were competitive with local drinking water prices [88]. However, transport was not yet accounted for in the calculation, and it is foreseen that the project requires larger amounts of treated water for true economic feasibility.

### 3.3. Industrial Wastewater Treatment

#### 3.3.1. Heavy Metal Removal

Heavy metals in water are a major concern around the globe due to their persistence and toxicity. Most of them can cause multiple organ damage, even at lower levels of exposure and are classified as either “known” or “probable” human carcinogens [89]. A number of these heavy metals are registered on the Substance Priority List (SPL) of the American Agency for Toxic Substances and Disease Registry, with arsenic, lead and mercury ranked on the first three positions [90]. The SPL indicates substances with significant potential threat to human health due to their known or suspected toxicity. Industrial processes all over the world produce wastewater with substantial concentration of heavy metals, which is then directly or indirectly, via a treatment plant, released to water bodies and drinking water sources. In 2016, over 60% of the reported heavy metal emissions into water in Europe came from direct or indirect industrial releases, mainly from the chemical manufacturing, non-ferrous metal processing and the energy supply sector [91]. The increasing concentrations of heavy metals in global water bodies observed from 1972 to 2017 [92,93] indicate a worldwide threat for the environment as well as human health and have made the removal of heavy metals a top priority in industrial wastewater treatment processes. 

The performances of conventional NF membranes regarding heavy metals like arsenic [94], cadmium [95], chromium [96], copper [97,98], lead [99], nickel [100] and zinc [101] have been thoroughly investigated and Abdullah et al. provide a brief overview [102]. However, for many industrial waste streams it is expected that these cannot be directly treated with SW NF due to the high fouling potential of these streams. Several HF NF membranes have proven to be a powerful treatment method since multivalent ions are retained effectively. Gao et al. designed HF NF membranes by adsorption of negatively charged chelating polymers poly(acrylic acid) (PAA) on a positively charged poly(ethyleneimine) (PEI) cross-linked P84 support [103]. The resulting rejections of the PAA adsorbed membrane for lead, copper, nickel, cadmium, zinc, chromium and arsenic were 94% or higher while a permeability of around 1 L·m^−2^h^−1^bar^−1^ was achieved. Tests on heavy metal mixtures confirmed an average rejection of 98%. Additionally, it was shown that the chelating polymers help enhance rejections through adsorption of heavy metal ions. The same research group investigated the performance of thin-film composite HF membranes made of blended sulfonated polysulfone (PS), PES and PEI and further modified with TMC [104]. The NF membrane delivered a pure water permeability of 5.1 L·m^−2^h^−1^bar^−1^ while retaining the heavy metal ions copper, nickel, and zinc more than 90% in single ion tests as well as in mixtures of those three cations (>95%).

Zhu et al. coated a polybenzimidazole (PBI) and PES bilayer on an HF PES/polyvinylpyrrolidone support for removal of heavy metal ions (cadmium, chromium, lead) from model wastewater [105]. The rejections of the NF membrane to magnesium and cadmium achieved 98% and 95%, respectively. By changing the pH of the solution, the rejections to chromium and lead reached more than 98% and 93%, correspondingly. In 2015, poly(amidoamine) dendrimer (PAMAM) was grafted on TFC HF NF membranes made of PES [106]. The modification successfully decreased the pore size and improved the pure water permeability, (around 4.0 L·m^−2^h^−1^bar^−1^ against 3.1 L·m^−2^h^−1^bar^−1^) attributed to an increased hydrophilicity, without affecting the rejection. For the grafted membrane, rejections for arsenic, cadmium, copper and lead reached more than 99%. In tests with ion mixtures, rejections of more than 98% for nickel, zinc and chromium were achieved. A pH dependency could be observed, indicating better removal rates at higher pH. 

Recently, a graphene oxide (GO)/ethylene diamine (EDA) bilayer NF membrane was investigated for heavy metal removal: Zhang et al. coated the GO/EDA network via the LbL approach onto the outside of a modified Torlon^®^ HF support [107]. The rejections for lead, nickel and zinc were tested as well as the long-term stability. The best results were reported for a 5-bilayer GO/EDA membrane with rejections higher than 95% for the mentioned heavy metals and a pure water permeability of 4.7 L·m^−2^h^−1^bar^−1^ at a pressure of 3 bar. During a 150 h-long performance test, permeability and rejection (only tested for lead) remained stable with a slight increase in water flux.

Wei et al. applied thin-film composite HF NF membranes on actual electroplating wastewater for heavy metal removal [108]. Membranes were prepared by interfacial polymerization of PIP and TMC on the HF PS/PES support. At 4 bar operating pressure, the rejection rates reached 96% for chromium, and 95% for copper and nickel and ion concentration was more than five times higher in permeate compared to the feed solution. Moreover, the membranes showed a good stability in the low pH (2.31) electroplating wastewater. 

Despite these several studies on the membrane development and the efficacy of these membranes on synthetic waste streams, little information can be found about the performance of HF NF on real wastewater streams. Such studies are required to assess the true applicability of these membranes for the removal of heavy metals from industrial streams. Upcoming research further needs to include strategies on how to deal with the concentrate streams coming from the NF process.

#### 3.3.2. Sulfate Removal

High loads of sulfates may damage the concrete in the sewage system in a so-called external sulfate attack [109]. Often, a massive formation of gypsum and ettringite formed during this external sulfate attack may cause concrete to crack and scale [110]. Additionally, under anaerobic conditions, sulphate can result in sulfuric acid which further damages the cement of the sewage pipes [110]. As a higher sulfate concentration is related to potentially higher damage [111], reducing the sulfate levels in wastewater can reduce the risk for sulfate attacks. To prevent irreversible damage to the sewage system, in the Netherlands the disposal limit of sulfate is set to 300 ppm [112]. Since NF removes bivalent ions like sulfate effectively, it is a well-suited technology for sulfate removal and has been reported for various applications, among others in mine drainage treatment [113,114,115], agricultural drainage treatment or the electronic manufacturing industry, where Jin et al. [116] investigated the performance of a commercial thin-film composite NF membrane (DK1812, GE) on a sulfate-rich wastewater from an electronic manufacturing plant. The initial sulfate concentration of 900 mg·L^−1^ could be reduced by more than 98% to 16 mg·L^−1^, enabling a direct water reuse as cooling water [116]. Furthermore, a recovery of 83% was realized proofing the high potential of NF. However, increased scale formation was detected at elevated concentration factors harming the process performance. Here, HF NF could be a suitable alternative because it enables for better scaling control and more effective cleaning methods as these membranes do not contain spacers around which scale growth is known to initiate [52]. 

In 2018, Jährig et al. upgraded the commercially available HFW 1000 membrane (Pentair X-Flow) with specific extra coatings to increase the retention capabilities and investigated the performance for sulfate removal in a pilot-scale set-up on well water with elevated sulphate concentration [43]. Pilot operation at a flux of 22.5 L·m^−2^h^−1^ and a recovery of 75% resulted in sulfate removal of 67%. 

HF NF membranes have recently been evaluated for sulfate removal in a German joint research project [117,118]. In the SULEMAN project, commercial HF UF membranes (Multibore^®^, DuPont inge GmbH) were coated via the LbL-approach to create NF separation properties [119] based on PDADMAC and PSS. The novel membranes achieved a MgSO_4_ retention of 90% and a pure water permeability of 15 L·m^−2^h^−1^bar^−1^ was reported. First tests on water with elevated sulfate amounts showed promising results of 80 to 90% sulfate removal and stable performance. 

#### 3.3.3. Organics Removal

Industrial wastewaters usually contain a diverse range of organic compounds in different concentrations. A lot of these substances can have a severe impact on human or environmental health, e.g., being carcinogenic or bioaccumulating in animals. Typical sources of organically polluted wastewaters are, among others, the petrochemical industry, textile industry, tanning industry, pulp and paper industry, rubber and tire production, chemical production, food and beverage industry and the energy supply sector [91,120]. We will show that, due to their robustness and high separation efficiency for small organic pollutants, HF NF has proven to be a successful technology for organic removal from industrial wastewater in single step processes [28,121,122,123,124,125].

##### Textile Industry

One important industrial sector is the textile industry. Since it produces a lot of wastewater loaded with complex mixtures of dyes, a treatment prior discharge is crucial to prevent damage to ecosystems and human health and align with legislations [126,127]. HF NF has been studied intensively in the textile industry and has shown great potential in effectively removing organic dyes from textile wastewater, while retaining low amounts of monovalent salts resulting in beneficial process conditions. 

Sun et al. fabricated a thin-film composite HF NF membrane by interfacial polymerization of hyperbranched polyethyleneimine and isophthaloyl chloride on a Torlon^®^ polyamide-imide dual-layer HF substrate [128]. The membrane rejected Safranin O and Orange II effectively (>99%), while a flux of 23 L·m^−2^h^−1^ and 20 L·m^−2^h^−1^, respectively, at a pressure of 5 bars was achieved. Shao et al. developed a thin-film composite HF NF membrane via interfacial polymerization of m-phenylenediamine (MPD) and PIP with TMC on a polyetherimide HF support [129]. The resulting membrane achieved a flux of 17.5 L·m^−2^h^−1^ at 6 bars and showed rejections as high as 90% and 98% for Safranin O and Aniline blue, respectively. 

Zheng et al. investigated the performance of a submerged thin-film composite HF NF membrane on biologically treated effluent from a textile industry [121]. The selective layer was formed by coating sodium carboxymethyl cellulose onto the outer surface of a polypropylene (PP) support. The submerged HF NF achieved a chemical oxygen demand (COD) reduction rate of 92% and color removal of 99% and exhibited a flux of 5 L·m^−2^h^−1^ at a transmembrane pressure of 0.8 bar and a concentration factor of 4. Furthermore, the membrane showed low salt retentions enabling the process to be operated at more beneficial process conditions.

Ong et al. performed pilot-scale studies with a polyamide-imide based HF NF membrane on textile wastewater [125]. The wastewater was a complex mixture of textile dyes, suspended solids, mineral oils, electrolytes and surfactants with very high COD (ranging from 3000 to 8000 ppm). During a 45 day period the membrane module showed excellent COD rejection rates (>95%). The initial flux of 3 L·m^−2^h^−1^ at 5 bars decreased significantly over time but could be regenerated after chemical cleaning several times during testing without influencing the COD rejection. 

A polysulfone-based HF NF membrane was prepared via interfacial polymerization of MPD and TMC by Mondal et al. [124]. The membrane performance was then investigated by treating textile wastewater from an Indian textile industry. Dye rejection for cibacron yellow, cibacron red, cibacron black and basic blue were 98% or higher and COD removal was 75–90%, thereby achieving the local permissible limit. The flux ranged between 4 and 11 L·m^−2^h^−1^ for various operating conditions. 

Ji et al. fabricated loose HF NF PSF/GO membranes by one-step non-solvent induced phase separation method without post treatment [130]. The membrane was evaluated on simulated textile wastewaters consisting of various congo red dye and NaCl concentrations. It exhibited excellent dye rejection (>99%), while NaCl rejection was below 5%. Moreover, the reported flux was in the range of 74–95 L·m^−2^h^−1^ at operating pressure of 2 bars. Recently, the same research group developed polyvinylidene fluoride (PVDF) HF NF membranes based on a solvent-free process [131]. The unique multilayer structure was constructed by deposition of graphene oxide and polypyrrole on the supporting layer. The membrane showed a high rejection for congo red (99.5%), direct yellow 24 (99.3%), acid orange 10 (99.1%) and rhodamine B (94.1%), whereas NaCl retention was as low as 4%. Moreover, a flux of 9 L·m^−2^h^−1^ was achieved at a pressure of 1 bar.

##### Oil and Gas Industry

Another important industrial sector is the oil and gas industry. In 2019, more than 12 million barrels of oil were produced per day in the US alone [132]. Huge amounts of wastewater are generated during oil extraction, since on average one barrel of oil results in three barrels of so-called produced water [133]. The composition of produced water can vary widely and depends among other things on location, extraction method or type extracted. Produced water typically includes following major groups: Water-soluble low molecular weight organic acids and monocyclic aromatic hydrocarbons, dissolved and dispersed oil, grease, production chemicals (e.g., corrosion inhibitors, biocides) and dissolved minerals [133,134]. Due to the high pollution and amount of generated produced water, sufficient treatment is necessary to meet the environmental regulatory requirements. Several physical, chemical and biological methods or combined technologies are proposed for produced water treatment [134]. Compared to the conventional methods, membrane filtration offers several advantages, including high quality permeate, low footprint, more automation, no need for extraneous chemicals and lower energy demand [135]. NF for produced water treatment is especially interesting as it operates at lower pressure than RO and, compared to UF, it has higher organics rejection [136]. 

Xu et al. studied the performance of three commercial NF membranes on produced water from a sandstone aquifer, namely NF-90 (Dow/Filmtec), TFC-S (Koch) and ESNA (Hydranautics). Among those, the NF-90 had the best TOC rejection with 87.6% and was ranked highest regarding the adjusted specific fluxes [137]. Mondal et al. tested the commercially available membranes NF270 (Filmtec) and NF90 (Filmtec) on produced water [138]. It was shown that the TDS concentration was reduced from 2090 ppm to 1780 ppm and 1340 ppm for NF270 and NF90, respectively. Further, TOC could be reduced from 136.4 ppm to 98.1 ppm and 89.7 ppm for NF270 and NF90, respectively. In 2013, Alzahrani et al. studied the performance of a highly hydrophilic NF membrane supplied by AMFOR INC^®^ on produced water. The membrane achieved a permeate quality that met 96% of the 74 measured parameters of WHO and United States Environmental Protection Agency quality standards for drinking water and for reuse as indirect potable water [139]. In a second study, they evaluated the toxicity of the obtained NF permeate [140]. While the TOC level was reduced by 48%, toxicity tests showed that the permeate surprisingly was still toxic which was attributed to unknown substances. 

All these papers reported membrane fouling as a major challenge in produced water treatment via NF. Here, HF NF could be a better alternative to the aforementioned SW membranes, as HFs can be cleaned more effectively (e.g., backwashing) and are less prone to fouling due to the absence of feed spacers, which have a considerable influence on fouling behavior [141].

Liu et al. fabricated a thin-film composite NF membrane via interfacial polymerization on a dual-layer (PES/PVDF) HF substrate [123]. The novel HF membrane has a permeability of 16.6 L·m^−2^h^−1^ and was tested on secondary effluents obtained from a petrochemical industry plant pre-treated by UF. A TOC removal of 97% down to 2 ppm was achieved while the TDS load was reduced by 39.6% with 633 ppm remaining in the permeate, which allowed for direct discharge into the local sewage system or to surface water. 

Recently, Virga et al. developed a stable HF NF membrane for produced water treatment. By cross-linking their LbL-coated poly(allylamine hydrochloride) (PAH)/PSS multilayers with glutaraldehyde, surfactant stable membranes were achieved [142]. The membrane showed excellent oil removal (>98%) for two synthetic produced waters and TOC retention of 96.5% and 83% for a cationic containing surfactant and an anionic containing surfactant, respectively. More important, flux recovery after cleaning was fully achieved for the cationic solution (100%) and it was possible to recover 80% of the initial flux while using the anionic surfactant solution. For both cases high removal rates were achieved and good cleanability was demonstrated, supporting the expectations that these membranes are a promising alternative.

##### Other

Wastewater of the soy sauce production process has a high chemical oxygen demand [143]. Before discharging it into the environment it is desirable to remove the color originating from caramel pigments and melanin or melanoidin and to lower the COD. Jang et al. showed the successful treatment of this waste stream by a combination of NE-70 NF membranes and a H_2_O_2_/O_3_ process, resulting in 98.1% color removal and a reduction of 98.2% of COD.

### 3.4. Ultrapure Water Production

**Figure 6:**
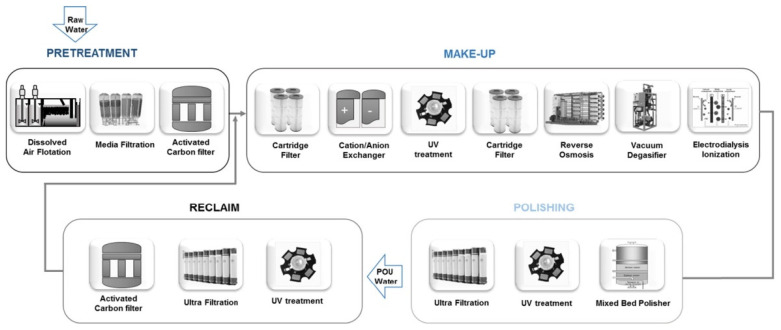
Schematic flow of a complex UPW production system for microelectronics manufacturing. A typical hybrid membrane treatment plant is presented which involves a combination of RO-MBDI-UF to provide microelectronics grade UPW. Reprinted from [145], with permission from Elsevier.

In several industrial applications the highest purity of water is needed in the production process. The total global market for ultrapure water was estimated to be USD 5.33 billion in 2016 and is projected to reach USD 10.31 billion by 2025 [144]. In, e.g., semi-conductor, pharmaceutical, photovoltaic and microelectronic industries contaminations to the lowest level are still detrimental in the production process. Therefore, several treatment schemes have been developed consisting of multiple different unit operations [145], and membranes often play a crucial role [146] (See Figure 6). A common process for applications in the semi-conductor industry uses both RO and tight UF membranes. The RO removes most of the contaminants, while a tight UF membrane (with an MWCO of around 10 kDa) serves as a final polishing step to remove the last pathogens and micro- and nanoparticles that end up in the permeate of the RO. Part of these contaminants in the RO permeate can even be a result of leaching out of the membrane modules. In order to assure a clean permeate after UF polishing, HF UF membranes are the preferred option, as they allow the best assurance of a clean permeate side. Typically, these modules are operated in an outside–in configuration.

Currently, even higher water qualities are being requested [147], which means that, among others, NF membranes can be introduced to substitute the final UF polishing step. Here especially the recent development on HF NF membranes is relevant, to enable clean permeate control just as in the UF membranes. Additionally, NF membranes can be applied when reclaimed water is used as the primary source in the ultrapure water process. The higher organic load of reclaimed water puts a strain on the current treatment schemes [147], but this can be relieved by NF processes. Currently, little attention is given to the application of NF in the ultrapure water processes, but this is expected to change in the future.

### 3.5. Food and Beverage Industry

In the previous sections, we discussed the (potential) applications of HF NF membranes with the focus on clean water recovery. In the following sections, we shift the focus towards the use of HF NF membranes in different industries for the recovery and reuse of valuable components or solutes. 

Specifically, the food and beverage industry is an emerging field for the use of NF membranes, and the first developments are starting in the dairy industry [148]. NF membranes can be used as a standalone system, but they are also often applied integrated with other membrane processes [149]. In the following paragraphs various food and beverage industries are covered and a variety of applications of NF membranes are discussed. 

#### 3.5.1. Dairy Industry

The dairy industry is one of the largest food processing industries in the world [150]. Many by-products are formed that can lead to problems of their use or management. Whey is a prime example by-product from the production of cheese [151]. Large volumes are produced, and due to the high organic content whey disposal is difficult. Additionally, it also still contains valuable resources that can potentially be recovered. For example, it can serve as a source of lactose and proteins that in turn can be used not only in the food and dairy industries, but also the pharmaceutical industry [151]. Recovering these compounds also lowers the COD and biological oxygen demand levels in the wastewater, which eases wastewater treatment prior to discharge. Das et al. showed that proteins and lactose could be recovered by using a combination of UF and NF [151]. UF was used to concentrate the proteins in the retentate. The permeate was then led to the NF membrane to concentrate the lactose. 

Next to proteins and lactose as valuable products, whey often also contains minerals at concentrations that are undesirable for applications [152]. Therefore, the concentration of whey is often combined with demineralization. NF membranes take care of partial demineralization and can be combined with an anion-exchange pre-treatment to reach a higher demineralization of whey.

Lactose can be a main ingredient in the production of other valuable products. One of them is galacto-oligosaccharides (GOS), resulting from the trans-galactosylation reaction of lactose. The GOS are of interest because of their prebiotic nature and are increasingly used in infant foods and functional foods. The final product of the synthesis contains, besides the GOS, mono- and disaccharides that reduce the prebiotic function. Therefore, it is key to remove these carbohydrates. In a study by Michelon et al. it was indicated that NP030 NF membranes can be used as a preliminary technique for GOS purification with a recovery of 61% [153].

#### 3.5.2. Sugar Industry

The production of sugar is one of the most energy-intensive processes in the food industry [154]. In order to reduce the energy consumption membrane processes have been intensively investigated as an alternative to traditional technologies. However, these applications can be challenging, especially due to the viscosities and high osmotic pressures of the solutions [155].

The removal of colorants from syrups is a key step in the sugar production process, because the final product, sugar, must meet strict standards and the color content should be as low as possible [154]. Usually the removal of non-sucrose compounds is done via liming and carbonation, followed by filtration—operations that are resource intensive and accommodate environmental pollution problems [154]. Gyura et al. showed that NF membranes can be of interest as an alternative method for the use in the separation of colored matter from green syrup [156]. Using a flat-sheet polyamide TFC membrane with an MWCO of 500 Da they proved to be able to decrease the color by 76%.

Another application is the use of NF membranes for the recycling of (anion) exchange resin regeneration effluents. The resins are mainly used to remove high-molecular weight colorants and must be periodically regenerated. This produces a waste brine with high amounts of sodium chloride, organic matter and COD. Salehi et al. investigated the use of polyamide tubular NF membranes for the recovery of usable brine [157]. They showed that brine could be recovered, leading to a reduction in water consumption of 90% and reduction in salt consumption of 77% for the regeneration process. Next to this, the colorants were removed for more than 99%.

Oligosaccharides are widely used as ingredients or additives in the food industry, owing to their nutritional and health function. In order to be used, the oligosaccharides should be separated from the monosaccharides present in reaction mixtures. NF membranes can be used for the purpose of purification and separation of oligosaccharides. It is important to note that for these types of processes, microbial growth can be a serious issue. Pruksasri et al. evaluated the use of NP030 NF membranes for the purification of GOS [158]. Microbial growth could be reduced compared to normal operating conditions by purifying at either 5 °C or 60 °C, where the lower temperature was more advantageous in terms of product yield. A product purity of 85% could be achieved and oligosaccharide recovery of 82%. With PEM NF membranes, oligosaccharides could also be separated, as was shown by Shi et al. [159]. The composite NF membranes resulted in 100% oligosaccharide retention and 63% glucose rejection, with a selectivity of maltose/glucose of 46. 

In 2016, Malmali et al. investigated the fractionation of sugars by PEM NF membranes [160]. The membranes were prepared by LbL coating of PDADMAC and PSS on a polysulfone support. The resulting membranes had a sucrose to glucose selectivity higher than 11, showing the potential of fractionating di- and monosaccharides with PEM NF membranes.

#### 3.5.3. Beverage Industry

NF membranes are also gaining increased attention in the beverage industry, especially for the alcoholic beverage industry, in order to control the alcohol content in beverages, but also for juice concentration [149,154]. 

**Figure 7:**
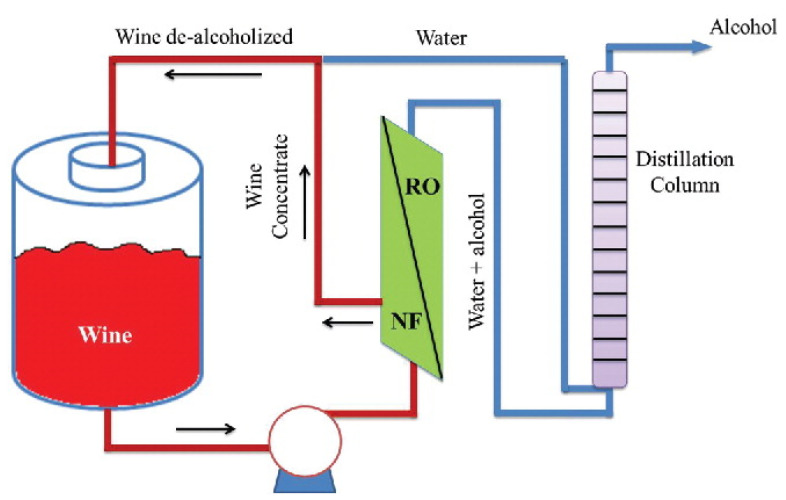
Process of wine de-alcoholization by coupling reverse osmosis or nanofiltration and distillation. Reprinted from [163], with permission from Taylor and Francis Ltd., http://www.tandfonline.com (accessed on 14 October 2021).

Increased interest for NF membranes is observed for the production of low alcohol content wines [149,161]. An important benefit, compared to thermal heat-based processes, to remove alcohol, is that volatile aroma compounds are not lost. A study from Catarino et al. comprises RO and NF membranes (including HF membranes) that were used for the removal of ethanol from a 12 vol.% red wine [162]. YMHLSP1905 NF membranes from Osmonics and NF99 and NF99HF NF membranes from Alfa Laval showed very promising results, with a good permeability to ethanol, while retaining the aroma compounds. Other tests were performed by first using pervaporation to recover aroma compounds that were later added to the dealcoholized wine, resulting in a better flavor sensation. It must be noted, however, that the final product still contains 8.5 vol.% of alcohol [162]. El Rayess and Mietton-Peuchot identified the opportunities (e.g., see Figure 7) and advantages of NF and RO in oenology, but highlighted the fouling issues typically observed in these applications [163].

An emerging issue in wine-making is the early ripening of grapes, a consequence of global warming, leading to a higher sugar content that results in an alcoholic content that is higher than desired. For this reason, research has focused on controlling the sugar content in grape must. The concept was proven by Salgado et al., who showed the successful reduction in sugar content of red must, using flat-sheet NF membranes [164]. 

Juice concentration is a process that helps to reduce the bulk volume and shipping costs of juices. Traditionally it is done by an evaporation process; however, this has some disadvantages such as loss of fragrance, taste and color. Membrane technology provides an alternative technique that allows maintaining flavors and aroma. Warczok et al. indeed showed that apple and pear juices can be concentrated by the use of NF membranes [165]. In other studies, the concept was also successfully applied for the concentration of blackcurrant and strawberry juices [166,167]. Furthermore, NF can also be applied to separate and purify phenolic compounds from pomegranate juice [168]. 

Coffee is another type of beverage for which NF membranes potentially can be of interest. Pan et al. showed that NF-2 SW NF membranes are capable of concentrating coffee extract. In another study, Ong et al. demonstrated the concept of decaffeination with the use of HF NF membranes (MWCO 470) [169]. Early results showed reduction of 25% in caffeine, with a volume reduction factor of 1.2. 

#### 3.5.4. Other

There are many more food industries besides the dairy, sugar and beverage industry where NF membranes can (potentially) be successfully applied. One of them is the production of tofu, with whey as a by-product. The tofu whey can be used as fertilizer but is also often discarded. However, this waste contains isoflavones that are a source of antioxidants. A combination of freeze drying and concentration via NF (PVDF SW) was used by Benedetti et al. to show the potential of using this process to recover valuable products from tofu whey [170]. 

Another application is the production of starch, where the NF membranes can serve as an outcome for the recovery of high-value proteins from potato starch wastewater. The potato proteins have a high nutritional value and antioxidant and functional characteristics that can be transferred into valuable food ingredients. Li et al. investigated the potential of HF UF and NF membranes for this purpose [171]. The polyamide TFC HF NF membranes could reject low molecular weight potato proteins for 92.1% combined with a COD rejection of 86.8%. Fouling resulted in flux decline over time, and could be recovered via cleaning to 84.7%. 

The above examples highlight the wide range of different food and beverage related applications possible for the use of (HF) NF membranes. While not all examples given here are HF configurations, it can be expected that HF NF will be used for these applications in the future as well, especially because of its benefits compared to SW NF when dealing with higher amounts of suspended solids. With the increasing need for smart use of materials and challenging new questions HF NF membranes can play a role in this.

### 3.6. Chemical and Petrochemical Industry

In the chemical and petrochemical industry, separation processes play a paramount role, accounting for 40–70% of the capital and operating costs [172]. For a variety of applications HF NF membranes can be or are already applied. The applications concerning the production of clean wastewater are already discussed in Section 3.3, and here we focus on the recovery and reuse of valuable products from process (waste) streams.

#### 3.6.1. Caustic and Acid Recovery

The recovery of acidic and alkaline solutions is an important application in the chemical and petrochemical industry, but also in many other industries such as the food industry. In most cases, they are used for cleaning purposes of apparatuses and production equipment via a so-called cleaning in place (CIP) process that consists of multiple steps and involves both alkaline, often NaOH, and acidic, often HNO_3_, solutions. These solutions are used in large quantities and substantially contribute to the total cost of the process. For example, in the dairy industry, per 1 L of treated milk, 0.2–2.0 L of acidic and alkaline effluents are produced [173]. By recovering and reusing these products, the process could be operated in a more ecologically and economically efficient manner [174]. 

A simple method to recover used cleaning solutions is to store the solution in a tank for a certain time and let the suspended material settle out [175]. The top part of the solution will be partially clarified and can be mixed with fresh cleaning solution to reach the desired properties and used again. However, dissolved and small compounds will not be removed and therefore the final quality of the solution will be relatively low. The quality of the recovered solution can be significantly improved by using an NF process.

##### Cleaning Solutions

Novalic et al. studied the recycling of sodium hydroxide by a tubular NF membrane (MPT-34) in the dairy industry. They showed that it is possible to recycle NaOH and that the quality of the recycled caustic soda is highly dependent on the starting conditions [174]. Santos et al. showed that also in petroleum refineries, the recovery of spent caustic by NF membranes can contribute to the overall performance of an industrial installation [176]. Besides being suitable for the recovery of alkaline solutions, Novalic et al. also showed that recycling acidic cleaning solutions is possible with NF membranes [177]. Here, it is important that the acidic cleaning solution should be applied after a caustic cleaning step (as is done in most cases), because otherwise the high concentrations of salts will lead to high osmotic pressure and the recovery will not be economically feasible. 

With time, single-phase detergents are gradually replacing the conventional acids and alkalis [178]. By doing so, the alkaline, acid and disinfection stages are integrated and the lead time and use of chemicals is reduced. However, these single-phase detergents are quite expensive and therefore also here the recovery is of interest. Fernández et al. showed the successful recovery of contaminated single-phase detergent from a CIP system in a yoghurt industrial plant using Koch MPS-34 membranes [178]. Furthermore, Kowalska et al. studied the recovery of acid single-phase detergents by different UF and NF membranes (a.o. tubular polyamide AFC30 and AFC80 NF membranes) [173]. It was shown that the spent acidic single-phase detergent could be recovered. For the AFC30 NF membranes, high protein retention (99.8%) and lactose retention (98.7%) was observed. At the same time the surface tension of the permeate increased, which might have a weakened effect on the detergency properties. 

##### Other Applications

Besides cleaning applications, sodium hydroxide is also used for other purposes, such as for the extraction of hemicellulose from wheat bran and barley husks. The hemicellulose is in turn used for the production of food packaging. Arkell et al. showed that the sodium hydroxide could be recovered using an NF membrane and that the pay-back time is expected to be less than two months [179]. 

The recovery of acids is not solely limited to cleaning solutions. There are many other examples of acids that are valuable enough to be recovered. Recent research showed that PEM HF NF membranes (dNF40 NX Filtration) can also be used for the separation of volatile fatty acids (VFA) from anaerobic effluents [180]. The recovered VFAs can be used not only in the chemical industry for the synthesis of, among other things, ketones, esters and alcohols, but also in the textile industry. By comparing four different separation methods (NF, RO, forward osmosis and supported ionic liquid membranes), the study showed that NF provides the highest permeance and allows for adequate selectivity between different VFAs. In another study, the recovery of succinic acid, as a platform chemical, from bioethanol waste was investigated and the potential for flat-sheet NF membranes was demonstrated [181]. In a similar way, carboxylic acids and phenols could be concentrated and recovered from hydrothermal liquefaction wastewater [182]. 

The above examples show that NF membranes can be successful for the recovery and reuse of both alkaline and acidic solutions in several applications. Still, there are only a limited number of acid and alkaline stable NF membranes commercially available that can be used for these applications. Conventional polymeric NF membranes (often polyamides) are not stable, especially under alkaline conditions [183]. Therefore, research is being performed to expand the range of membrane materials suitable for the more demanding conditions. In a recent paper by Elshof et al. it was shown that PEM HF NF membranes can have long-term stability in both extreme acidic and alkaline solutions [184]. For this reason, it is expected that these types of membranes will also be interesting candidates for the above-mentioned applications.

#### 3.6.2. Metal Recovery

The composition of green energy supply technologies like wind, solar or hydrogen systems is significantly more material intensive than current traditional fossil-fuel-based energy supply systems [185]. As these technologies will very likely be necessary to reach the goals of the Paris Climate Agreement and to ensure a transition to a low carbon economy, the world’s demand of metal resources will rise during the century and pressure on current supplies might increase [186]. Among others, it is expected that the worldwide demand of zinc, lead and copper will exceed available resources by the middle of the 21st century [187]. Therefore, recovering metals from water or wastewater sources will become more and more important to satisfy rising demands. In 2013, Qin et al. investigated the recovery of CuSO_4_, ZnSO_4_, NiCl_2_ and CdCl_2_ from artificial wastewaters by using HF NF membranes [188]. A modified polyacrylonitrile (PAN) UF substrate was coated with bilayers of PEI and PSS, resulting in a positively charged NF membrane. Rejections of 98.0% for CuSO_4_, 95.5% for ZnSO_4_, 95.7% NiCl_2_ and 94.9% for CdCl_2_ were achieved at salt concentrations of 500 mg·L^−1^, along with permeation fluxes between 19 and 24 L·m^−2^h^−1^. 

One metal of high interest is lithium, which is commonly used in Li-ion batteries. Due to their versatile applications in, e.g., plug-in hybrid vehicles, in consumer electronics and in space, military and medical applications, the worldwide lithium consumption for 2025 is expected to be twice as high as it was in 2017 [189]. To keep up with the world’s increasing demand, recovery of lithium from water sources or lithium-containing wastewater streams might become more and more attractive in the future. Major challenges for lithium recovery are the low concentration and the separation from many other alkali metals [190]. Here, HF NF membranes can be applied for separation as they can offer an effective barrier for divalent ions. The successful separation of an aqueous MgCl_2_/LiCl mixture was shown by applying a positively charged polyamide composite HF NF membrane [191]. The membrane was produced by interfacial polymerization of 1,4-Bis(3-aminopropyl) piperazine and TMC on a PAN HF support membrane. Lithium concentration in the permeate was reported to be more than three times the feed and the MgCl_2_/LiCl ratio changed from 20 in the feed to 7.7 in the permeate. Similarly, Zhang et al. recovered lithium from an artificially created salt lake brine by positively charged three-channel HF NF membranes [192]. For this synthetic solution (MgCl2 1866 ppm and LiCl 134 ppm), a flux 34 L·m^−2^h^−1^ was reported. The rejections of MgCl_2_ and LiCl were 95% and 18%, respectively, while the MgCl_2_/LiCl ratio could be reduced from 21.4 to 1.3. However, salt rejection and flux both decreased when a solution of CaCl_2_, MgCl_2_, NaCl and LiCl was used. MgCl_2_ rejection was lowered to 78% and LiCl rejection was reported at 15%, leading to a MgCl_2_/LiCl ratio of 3.0 in the permeate. However, the generally low concentrations of lithium make these processes not economically attractive, yet. The future demand and availability of lithium will determine its feasibility. 

Another metal of growing interest is the relatively scarce and expensive rare earth scandium. Its aluminum alloy forms stronger, heat tolerant and weldable aluminum products, e.g., enabling to apply weldable parts that reduce aircraft weights by 15–20% [193]. Therefore, recovery of scandium from secondary resources is of importance to meet increasing demands. Remmen et al. applied a PEM membrane on a titanium dioxide pigment production waste for scandium recovery [194]. A PES HF UF membrane was coated with bilayers of PDADMAC/PSS and compared against a conventional acid resistant flat-sheet membrane. The LbL membrane showed superior results in regard to scandium retention (60% vs. 50%) as well as higher fluxes, with the LbL membrane reaching 27 L·m^−2^h^−1^, whereas the conventional flat-sheet membrane achieved a flux as little as 1 L·m^−2^h^−1^ under the same process conditions. Additionally, a better selectivity towards major impurities (e.g., iron) was achieved.

#### 3.6.3. Phosphorus Recovery

**Figure 8:**
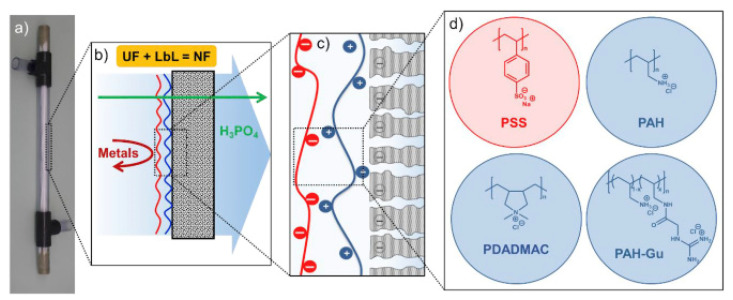
(**a**) Picture of a sulfonated sPES UF membrane from Pentair X-Flow, and schematic presentations of (**b**) alternately deposited PEs (only 2 PEs are given for matters of simplicity), including a summary of the separation properties, (**c**) a zoom-in of the interfaces inside the lumen, (**d**) molecular structures of the PES used in this study. Reprinted from [198], with permission from Elsevier.

Phosphorus is essential for food supply, mainly because of its use as fertilizer [195]. The element is becoming scarce and therefore phosphorus recovery has become increasingly important. Remmen et al. evaluated the use of PDADMAC/PSS PEM HF NF membranes for phosphorus recovery from sewage sludge ash [196]. For this, they used a 10% phosphoric acid solution (containing 2 g·L^−1^ aluminum) as model feed solution. Desired low retentions of phosphoric acid were obtained (<10%), such that a purified H_3_PO_4_ solution could be achieved in the permeate, with a phosphorus recovery of 75%. At the same time, impurities such as aluminum were retained >95%. The obtained permeabilities were significantly higher than commercial benchmark membranes (e.g., A-3012 AMS membrane). Later they investigated the stability of LbL based PEM membranes when immersing or filtering 15% H_3_PO_4_ solution [197]. Using PES as support layer, the membranes proved to be very stable after both immersion and filtration. LbL on SPES membranes showed a magnesium retention loss after immersion but were also stable upon filtration. Furthermore, it was observed that magnesium retention decreases with higher ionic strength solutions, but by altering process parameters, magnesium retention values up to 85% could be reached even at 500 mM Mg^2+^. Paltrinieri et al. studied the relation between membrane structure properties and phosphorus recovery (see Figure 8) [198]. Three different polycations (PDADMAC, PAH, Modified-PAH) were each combined with PSS as polyanion to form a PEM NF membrane. It was shown that PDADMAC/PSS membranes had the highest permeability and phosphorus recovery, yet lower multivalent metal retention as a result of its loose, less interpenetrated structure. Using PAH modified with guanidium groups, a higher retention was observed and therefore a less-metal contaminated permeate. However, the phosphorus recovery was lower. This was attributed to a more dense, compact layer. These examples prove that HF NF membranes can successfully be used for phosphorus recovery and it is expected that these will become an important solution for this purpose.

#### 3.6.4. Organic Solvent Nanofiltration

Organic solvent nanofiltration (OSN) is a promising technology for the purification and recovery of organic solvents [172]. Being able to recover and purify these solvents is an important step to limit the impact on the environment and save costs. Compared to conventional technologies, NF offers advantages such as operating at mild conditions, low footprint and lower energy demand [199]. There are several techno-economic evaluations that show that membrane filtration, either alone or in combination with traditional technologies, can give rise to a reduction in energy consumption, while maintaining the separation efficiency standards. On top of that, membrane filtration offers versatility, while it can be used for organic solvent concentration, purification and solvent exchange [172]. 

Despite the great potential for OSN membranes, their use is still limited. This is mainly because of concerns about the stability of the traditional polymer materials in these challenging conditions and limited fundamental knowledge of transport phenomena in OSN membranes [172]. The commercial OSN membranes used in industry are almost exclusively polymeric SW membranes, because of their ease of fabrication and modification [200]. HF membranes are not in commercial use yet, despite some of the advantages mentioned earlier [201]. Therefore, in recent years, research on OSN HF membranes has addressed these issues and has shown the potential of these membranes for treatment of organic solvents. 

There are many different materials suggested for the preparation of OSN HF membranes, often with the goal to improve the stability in organic solvents. The most commonly used materials for the preparation of OSN membranes are polyimide, polyacrylonitrile and polybenzimidazole. Goh et al. (2020) fabricated solvent resistant polyimide HF membranes [202]. Polyimide HF substrates were coated with a thin selective polyamide layer via interfacial polymerization. The resulting membranes showed to be stable in acetone and isopropanol and can reject acid fuchsin (585 Da) in acetone for 90%. Another preparation method involves the formation of a dense layer during phase inversion followed by chemical crosslinking [203,204]. Wang et al. showed the preparation of integrally skinned asymmetric polyimide based HF [204]. It was shown that the swelling degree and solvent stability of HF OSN membranes could be controlled by the shear rate of the spinning solution. The membranes maintained a high rejection for rhodamine B in ethanol after seven days immersion in dimethylformamide (DMF). 

Polyacrylonitrile HF membranes can also be used for OSN applications, as was shown by Tham et al. [205]. They showed that, by chemical crosslinking of PAN HF membranes, NF membranes could be obtained that showed excellent rejection (>99.9%) of remazol brilliant Blue R with a reasonable ethanol permeance. 

Robust polybenzimidazole (PBI) HF membranes for OSN were prepared by Asadi Tashvigh et al. [206]. Filtration tests with tetracycline/methanol and L-α-lecithin/hexane mixtures showed high rejections and permeances of 3.5 and 7.1 L·m^−2^h^−1^bar^−1^ for methanol and hexane, respectively, indicating an exciting potential for use in solvent recovery. Other polybenzimidazole membranes were prepared via a green cross-linking method, also showing good prospects for solvent resistant nanofiltration (SRNF) applications [207]. To save time, the crosslinking step can even be applied immediately during the phase inversion step [208]. 

Another type of polymer used for preparing OSN hollow fibers is polyaniline (PANi). Loh et al. demonstrated that intrinsically skinned asymmetric PANi hollow fibers are stable in organic solvents, such as DMF and acetone, and could be used for NF in acetone [209]. 

Recent research by Li et al. focused on the improvement of the selectivity of OSN membranes [201]. To this end Torlon^®^ HF membranes were prepared by tris(2-aminoethyl) amine and 4-sulfocalix[8]arene molecular engineering. The membranes showed excellent stability in DMF and were able to selectively sieve methylene blue over N,N-dimethyl-4-nitroaniline in methanol with a separation factor of 14.5. This study highlights the potential of these membranes to be carefully engineered, such that sharp molecular sieving capabilities can be obtained.

A completely different application is the use for liquid phase organic synthesis reactions, for example, for solvent exchange. Often, synthesis routes involve a sequence of different reactions. When in the first stage of the synthesis route a solvent is used different to the one used in the second stage, solvents are traditionally exchanged via distillation, but this can also be done via OSN. Livingston et al. showed the proof of principle of this technique with a flat-sheet polyimide membrane for the solvent exchange from toluene to methanol [210]. Similarly, they indicated the potential for homogeneous catalyst recycle.

The selective layer cannot only be prepared via interfacial polymerization or chemical crosslinking, the coating of polyelectrolyte multilayers is another successful method. Several researchers have shown the potential of different types of PEM NF membranes for use in SRNF applications [211,212,213,214]. For this, both strong and weak polyelectrolytes can be used. For example, Ahmadiannamini et al. showed the preparation of PEM NF membranes with the strong polyelectrolytes, PDADMAC and PSS, that showed retentions up to 99% for rose bengal in isopropanol (IPA) [215]. On the other hand, Ilyas et al. used the weak polyelectrolytes PAH and PAA, resulting in membranes that are stable long-term in IPA, acetonitrile and DMF [212].

The variety of examples shows that NF membranes have an immense potential for use in OSN applications. This includes not only the recovery of solvents and concentration of solvents, but also solvent exchange applications. Nowadays the standard is still focused on mostly SW NF membranes. While for most of the previously mentioned applications HF benefits from a better fouling control, this is typically not an issue in OSN. Therefore, only if HF could become beneficial compared to SW for OSN is it expected that a shift towards HF can be made. An example might be if the specific membrane area could be increased as compared to SW, but first the fiber integrity problems need to be tackled.

### 3.7. Biorefinery

A biorefinery is the processing facility that converts biomass into valuable products or energy that can replace fossil oil refineries. Different biomass materials can be treated, such as wood, straw, starch, sugars, waste and algae, with lignocellulose being the most abundant biomass on earth. The production of valuable compounds in a biorefinery is complex due to the variability of the composition of the biomass. Therefore, separation and purification processes play a critical role in biorefineries as these processes can be responsible for up to 50% of the capital operational costs. Pressure driven membrane filtration processes such as UF and NF have emerged as promising separation technologies in many applications of biorefineries and some examples are presented below. Currently, most of the studies have been carried out on more conventional membrane geometries, attributed to the limited availability of specific HF NF membranes. However, it is foreseen, especially based on the typical high TOC and TSS levels that characterize biorefinery processes, that HF NF membranes will play a significant role here.

Algae are a promising biomass to produce biofuel. Algal biomass harvesting membrane technology is seen as a promising technique, since it provides almost complete retention of biomass and offers a very economical competitiveness when compared to other more energy-consuming methods. Membrane filtration is minimally disruptive to harvested biomass, causes minimal stress to the algal cells and avoids chemical additives (e.g., flocculants or pH adjustments required for flocculation) that may otherwise degrade the quality of harvested biomass or bioproducts [216]. Bhave et al. studied the application of polymeric HF MF (Pall Corp., New York, NY, USA) to dewater microalgae for biofuel production achieving an energy reduction of at least 80% over traditional methods as centrifugation or froth flotation [217]. Additionally, these membranes also renovated growth media to acceptable levels for recirculation. The major drawback of membrane filtration is the low flux, especially at high biomass concentration. Therefore, several studies have suggested to combine membrane filtration as preliminary separation before centrifugation for microalgae harvesting [218,219,220].

Membrane technology can also be applied for the recovery of lignin from lignocellulosic biomass. Lignocellulose is composed mainly of cellulose, hemicellulose and lignin, which must be separated in order to process them into specific products. Cellulose and hemicellulose are biopolymers of sugars and thereby a potential source of fermentable sugars to produce biofuels. Lignin is a complex biopolymer rich in aromatic subunits that can be converted into high value bio-sustainable chemicals such as vanillin, antioxidants or polyamide. Biorefinery of lignocellulose involves pretreatment of biomass, processing and synthesis of biofuel or valuable products and finally purification. Lignocellulose biomass is hardly soluble in common solvents and its economic hydrolysis process into fermentable monosaccharides remains a major challenge. Common pretreatments are acid, alkali, organosolv (aqueous organic solvents like methanol, acetone, ethanol and ethylene glycol) or ionic liquids.

Ionic liquids have shown good results, but their reuse is required for the economic viability of the process. Most NF membranes are inefficient since sugars are noncharged, ionic liquids have a low charge density and both have similar molecular weights. Flat NF membranes fabricated by LbL deposition of charged polyelectrolytes (PSS and PAH), with controlled charge density and pore size, have been applied for the recovery of ionic liquid from dilute aqueous solutions containing monomeric sugars [221]. Selectivity above 50 was achieved for 1-butyl-3-methylimidazolium chloride and cellobiose. Lignin concentration from synthetic organosolv liquors by NF has been studied and lignin rejection of 99% has been achieved with flat thin film composite membranes, consisting of a polyamide selective layer on a commercial polysulfone support (FilmTec NF270 from Dow) [222]. Gomes et al. studied the concentration of synthetic mixture of phenolic compounds by commercial flat-sheet NF membranes (FilmTec NF270 from Dow and MPS-34 from KOCH) [223]. The mixture mimicked the composition of oxidized black liquor, and a significant concentration was achieved with high permeate fluxes and rejections above 90%. For separating hemicelluloses from process liquors containing sodium hydroxide, Schlesinger et al. showed that NF is preferable over UF for quantitatively retaining hemicellulose at molar masses above 1000 g·mol^−1^ [224]. High-value organic acids can be produced from fermentation of biomass and the primary challenge is the downstream recovery of them. NF has been employed for the recovery of succinic, lactic, butyric, acetic and fumaric acids [225]. Sosa et al. evaluated different NF membranes (Filmtec NF270 from Dow and NF-DK and NF-DL from GE Osmotics), achieving fluxes between 25 and 32 L·m^−2^h^−1^ and succinate rejections above 90% and reaching negative rejections for acetate and formate [226].

**Figure 9:**
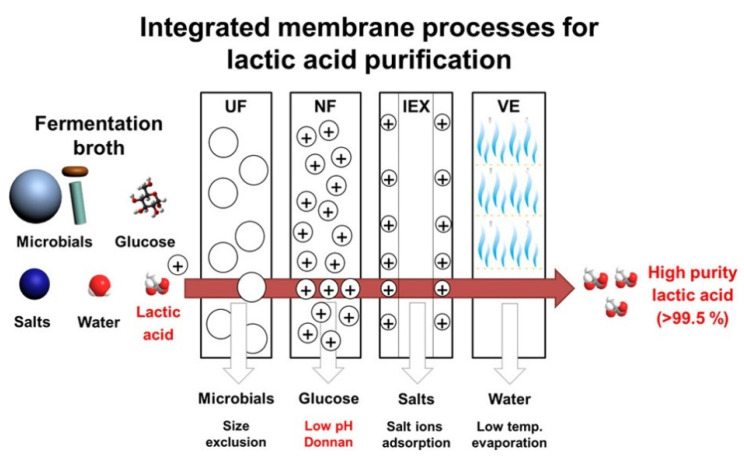
Integrated membrane processes for lactic acid purification. Reprinted with permission from [229]. Copyright 2017 American Chemical Society.

Another application of membrane technology is the separation of carboxylic acids from biorefinery solutions. Lactic acid is widely used in foods, detergents, pharmaceutical and cosmetic applications and in the chemical industry. Lactic acid is a naturally produced organic acid that can be commonly obtained by either chemical synthesis or carbohydrate fermentation. Lactic acid production by lactose fermentation bears high costs due to the separation steps required for food-grade lactic acid. To reduce the costs, different separation techniques have been studied, such as ion exchange, electrodialysis, distillation and membrane technology. In some studies researchers have shown that NF can be used to separate organic solutes (e.g., glucose and lactic acid) from fermentation broth [227,228]. Figure 9 provides a schematic of an integrated membrane process for the purification of lactic acid. Due to the difference in the molecular weights of glucose (180 g·mol^−1^) and lactic acid (90.08 g·mol^−1^), glucose can be effectively separated from lactic acid. The electrical charge of the NF membrane also affects organic molecule separation via Donnan exclusion effects [229].

The application of membrane technologies in biorefineries is expected to grow, but still only few studies using hollow fiber nanofiltration can be found. Since these membranes can handle better (bio)fouling—one of the main drawbacks in the application of membrane technologies to biorefineries—they would have the potential to change the economic evaluation of biorefinery downstream processes, making them industrially accessible.

## 4. Conclusions and Outlook

NF membranes offer solutions in a wide variety of different processes. The specific selectivity that the NF separation layer provides improves current or enables emerging applications. These include freshwater treatment, to remove hardness and natural organic matter, and municipal or industrial wastewater treatment, to remove harmful contaminants. In addition, NF can be employed to recover solutes or resources from (waste) streams, especially if NF selectivity is tuned or designed to the specific process. In many cases, high fouling rates or conditions of the feed limit the use of the traditional polyamide-based NF membranes. For such processes, a hollow fiber membrane geometry is beneficial and has been studied intensively.

Next to lab-scale tests, many studies have been carried out on larger pilot and demo-scales to prove the technical feasibility. This has led to commercial availability of HF NF modules that are now operated in full-scale installations, a testament that this membrane field will grow in the coming years. Given the variety of the streams and solutes, and the novelty of the different processes presented here, a better understanding or prediction of performance of NF membranes will be needed to accelerate the acceptance of these new membranes in the market.

## Figures and Tables

**Table 1:** HF NF membrane suppliers [23,24,25,26,27].

Company	Location	Product Name	MWCO (Da)	Operating Mode	Material Category
3E Memtech Pte Ltd.	Singapore	3E-NF20A 3E-NF40A 3E-NF60 3E-NF80A 3E-NF90A	n.a.	Inside-out	PES/PVDF + proprietary NF coating
De.mem Limited	Australia/ Singapore	De.mem NF	>200	Outside-in	PEI based TFC
NX Filtration	The Netherlands	dNF80 dNF40	800 400	Inside-out	PES-PEM
Pentair X-Flow	The Netherlands	HFW1000	1000	Inside-out	PES-PEM
Ochemate	China	NÜF N80	200–500	Outside-in	Polyamide based TFC

## Data Availability

Data sharing not applicable. No new data were created or analyzed in this study.
